# Giant splenic cyst: A case series of rare and challenging cases from the last 22 years

**DOI:** 10.1016/j.ijscr.2023.108263

**Published:** 2023-04-26

**Authors:** Kiki Lukman, Bambang Am Am Setya Sulthana, Deny Budiman, Prapanca Nugraha

**Affiliations:** Division of Digestive Surgery, Department of Surgery, Faculty of Medicine, Universitas Padjadjaran / Dr. Hasan Sadikin General Hospital, Bandung, Indonesia

**Keywords:** Case series, Cyst, Splenectomy, Splenic disease

## Abstract

**Introduction and importance:**

Splenic cyst is a rare disease, with reported incidences ranging from 0.07 to 0.3 %. A splenic cyst is typically discovered inadvertently and may not cause any symptoms until it grows to a significant size. In some cases, complications such as acute abdomen may be brought on by an intracystic hemorrhage, rupture, or infection. As a rare disease, diagnosing a splenic cyst is still precarious because only a few cases have been reported.

**Case presentation:**

The first case is a 23-year-old Asian man without a significant history of illness who complains of a left upper quadrant mass that he discovered 10 years prior. Since then, the mass had been gradually growing and had been accompanied by severe pain. Walking made the pain worse; lying down made it lessen. A computed tomography (CT) scan of the abdomen showed a 20.05 × 15.95 × 26.71 cm splenic cyst. Surgery for a peri-cystic splenectomy was done. A primary splenic cyst was identified in the specimen after microscopic and macroscopic examination. After 10 days, the patient was released from the hospital without complications. The second case is that of a 28-year-old Asian man who complained of a mass in their abdomen that was getting bigger in size. Four years prior to the complaint, the patient had fallen while driving a motorcycle, and the left side of his abdomen collided with the sidewalk. In this patient, a splenectomy—the complete removal of all spleen parts—was done. The specimen's macroscopic and microscopic examination revealed a splenic pseudocyst. The patient was discharged after three days without complications.

**Clinical discussion:**

Splenic cysts are considered rare and challenging to diagnose, as there have been only a limited number of reports. Nevertheless, proper management is still needed, as it carries the risk of rupturing and causing complications such as peritonitis and anaphylactic reactions. Considering the risk of overwhelming post-splenectomy infection (OPSI), conservative treatment can be the gold standard for splenic cysts. However, considering the risk from the size of the cyst, splenectomy or peri-cystic splenectomy is an appropriate surgery option for a splenic cyst.

**Conclusion:**

Splenectomy, or peri-cystic splenectomy, is a surgery option for a splenic cyst with significant size and rupture risk.

## Highlights

• Splenic cyst is rare.
• Partial splenectomy can result in recurrence and post-operative hemorrhage.
• Total splenectomy should be avoided due to the risk of sepsis.
• Conservative treatment is one of the gold standards for splenic cysts.

## 1 Introduction

A splenic cyst is a rare illness, with a reported incidence of only 0.07–0.3 % [1], [2]. Splenic cysts are classified as primary or secondary. Splenic cysts are divided into primary and secondary categories. Echinococcus granulosus is an example of a parasitic main cyst and a non-parasitic (congenital or neoplastic) cyst commonly referred to as a “true” or “primary” cyst. A secondary cyst is generally induced by trauma. A splenic cyst is usually found incidentally and shows no symptoms until it grows to a significant size. In some cases, complications such as rupture, infection, or intracystic bleeding may occur [1], [3], [4].

Symptoms of splenic cysts include nausea, vomiting, satiety, changes in gut function, persistent coughing, dyspnea, weight loss, left shoulder-referred pain, dyspepsia, epigastric abdominal discomfort, and pain in the left upper quadrant of the abdomen. Splenectomy used to be the standard treatment, but it is now being called into question, owing to the current trend toward conservative surgery, particularly for benign pathologies, as well as increased awareness of postoperative complications [5], [6].

As a rare disease, diagnosing a splenic cyst is still challenging because only a few cases have been reported. However, a splenic cyst must be diagnosed given the risk of spontaneous rupture or infection, which can result in acute abdomen [7]. Here we present the two cases from the last 22 years of splenic cysts that we found in a tertiary general hospital in West Java, Indonesia. The SCARE criteria were followed in reporting this case report [8].

## 2 Presentation of cases

### 2.1 Case 1

**Fig. 1:**
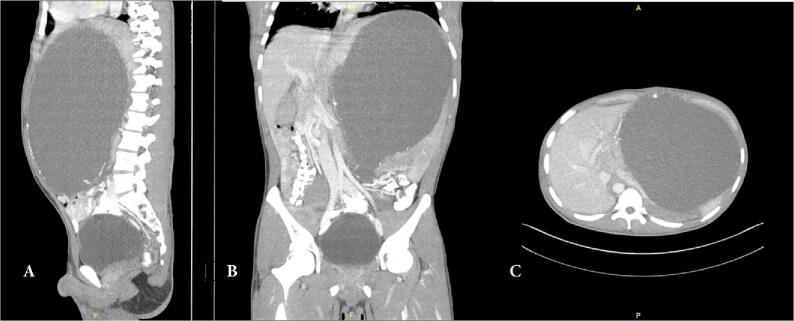
(A) The longitudinal plane (B) the coronal plane (C) and the transverse plane of a contrast abdominal CT scan showed a splenic cystic formation, rounded and with a well-defined border.

A 23-year-old Asian man with no major medical history complained about a mass he found 10 years ago in the upper left abdominal quadrant, but he seemed reluctant to undergo surgery and decided to seek conservative treatment. The mass was the size of an orange and had been slowly enlarging since then until it was as large as a football and was associated with sharp pain, so he sought medication. The pain was exacerbated by walking and relieved by lying down. He also suffered from early satiety and constipation. There were no signs of rectal bleeding, hematochezia, nausea, or vomiting. The patient had no prior history of abdominal surgeries, pancreatitis, alcohol abuse, gallstone disease, or trauma. Physical examination revealed a visible asymmetry in the bowel contour in the left upper quadrant. A well-defined mass was palpable below the left costal margin. There were no signs of abdominal tenderness or rebound tenderness on palpation. The contrast abdominal computed tomography (CT) scan revealed a 20.05 × 15.95 × 26.71 cm homogenous intraabdominal mass that was partially septated with well-defined regular edges in almost the entire abdomen, from the left upper abdomen to the middle, which pressed the surrounding organs (Fig. 1). He was diagnosed with a giant splenic cyst and scheduled for open surgery.

**Fig. 2:**
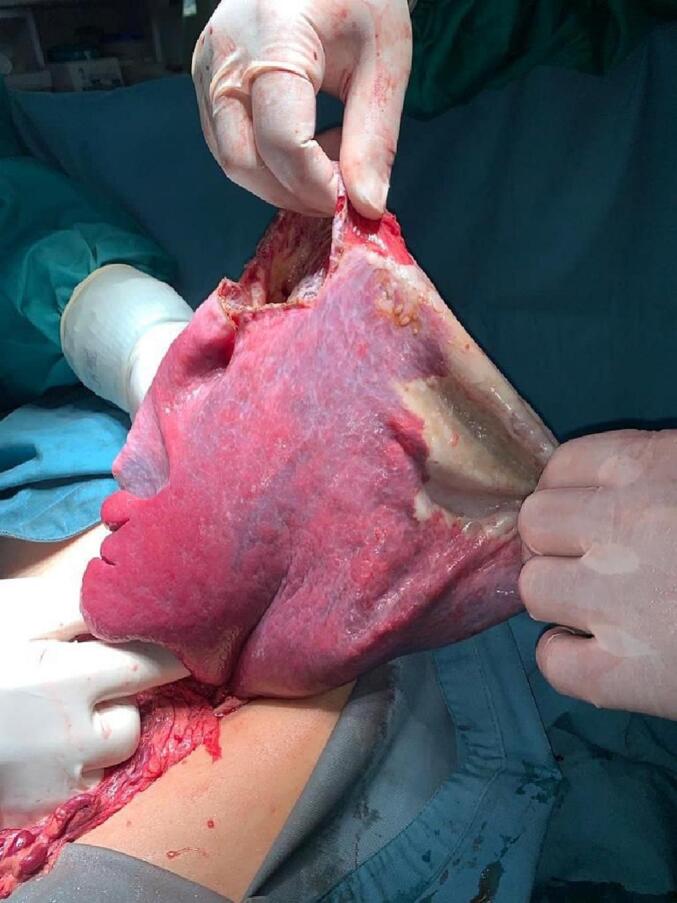
Splenic cyst after cyst fluid drainage.

**Fig. 3:**
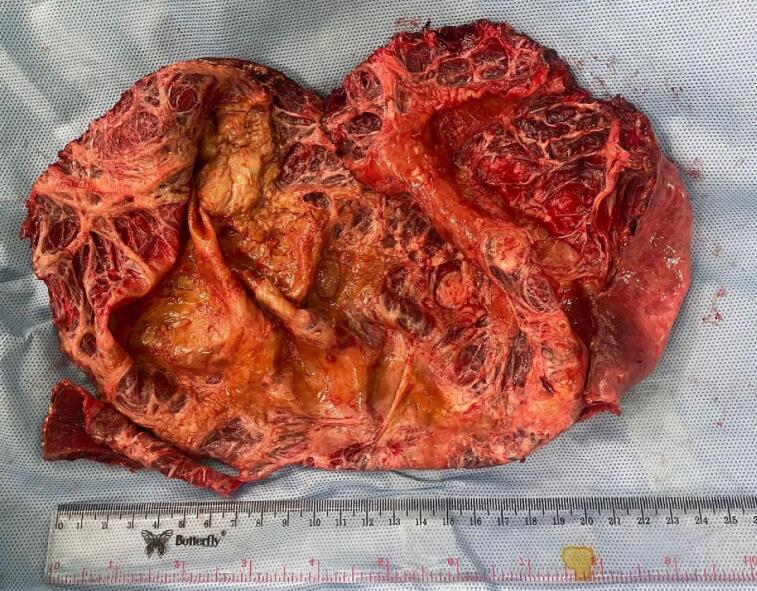
Cyst wall after peri-cystectomy.

One consultant gastrointestinal surgeon, one gastrointestinal fellow, and one surgical resident at a tertiary general hospital carried out the operation. Intraoperative findings showed that the cyst originated from the spleen. The cysts pushed against the left side of the stomach wall and the left lobe of the liver. An 5 cm incision was made in the cyst capsule using the monopolar cautery, and the cystic fluid was gently aspirated to keep leaking out into the abdominal space and so that the cystic capsule could be seen (Fig. 2). After the drainage of 6.5 l of brownish cystic fluid, four big pieces of gauze were inserted into the peri-splenic space. Then the cyst capsule was excised in a peri-cystic splenectomy using a harmonic scalpel; almost all of the capsule was excised (Fig. 3), and the rest of the capsule that adhered to the spleen was removed by mucosectomy using a monopolar cautery known as the “Lily technique” [9] to prevent further cystic fluid production. After removal of the gauzes and peritoneal toilet, an abdominal drain was inserted.

Postoperative care included maintenance of the fluid, antibiotics, a soft diet, and early mobilization. He was hospitalized for ten days for the observation of abdominal drain production, which was around 100 cc in the first week after surgery and diminished in the second week. One month after surgery, he went for a follow-up visit and was doing well, and there were no signs of complications such as abdominal pain, recurrence, or infection at the surgical site [10], [11]. When asked for his perspective on surgery, he said, “I'm happy that I'm getting better, and I should do the surgery earlier.”

**Fig. 4:**
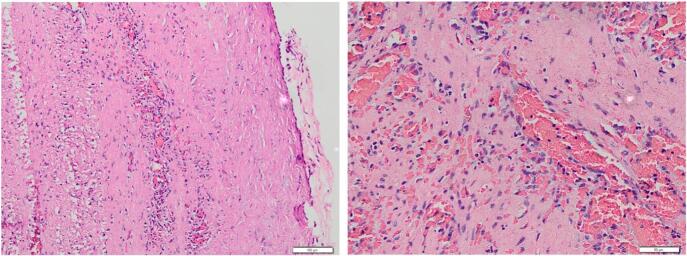
The microscopic view of the histological section shows that the cyst wall structure is lined with mesothelial cells, which are mostly erosive, and the nucleus is within normal limits (left picture). Beneath it, a stroma of fibrocollagen connective tissue is seen, which is partially hyalinized and covered with inflammatory cells, lymphocytes, and histiocytes, accompanied by dilation of blood vessels and bleeding. Also visible are red pulp and white pulp within normal limits. No sign of malignancy was seen (right picture). (For interpretation of the references to colour in this figure legend, the reader is referred to the web version of this article.)

Cyst fluid analysis was not performed as standard for a benign lesion, and the splenic cyst capsule was sent for histological examination. Anatomical pathology results in this patient showed a cyst wall structure coated by erosive mesothelial cells with a normal cell nucleus. Under those structures, there are fibro-collagenous connective tissue stroma, which has undergone hyalinization, lymphocyte cells, and histiocytes, accompanied by blood vessel dilatation and bleeding. There is also a normal white pulp. There is no sign of malignancy. The diagnosis for this patient's pathological condition is a primary splenic cyst (Fig. 4).

### 2.2 Case 2

This case was taken from the medical record in 2016, where radiologic imaging and intraoperative images were not available for this case. A 28-year-old Asian man arrived at the hospital's polyclinic for digestive surgery, complaining of a growing mass in his abdomen. He had a history of motorcycle accidents in which the left side of his abdomen collided with the sidewalk four years prior. The patient did not seek treatment from the hospital at the time and instead went to a traditional clinic. There were no reports of defecation difficulties or changes in defecation pattern, diarrhea, or bloody stools, and no reports of nausea or vomiting. An abdominal ultrasound was performed, which indicated a splenic cyst with a diameter of 12.7 cm. The abdominal CT scan data were not available in the medical record. A laparotomy for splenic cyst removal was scheduled. One consultant gastrointestinal surgeon, one gastrointestinal fellow, and one surgical resident at a tertiary general hospital carried out the operation. Intraoperative findings showed that the cyst originated from the spleen, was located deep within the spleen, and was surrounded by splenic parenchyma. An attempt for marsupialization and removal of the cystic capsule were not possible considering the possibility of bleeding, and a complete splenectomy was decided. An abdominal drain was inserted. He was hospitalized for three days before being discharged. An edematous fibro-collagenous connective tissue stroma with lymphocyte cells was revealed by histological examination. There were a few cystic cavities between those tissues, with no epithelial cells on the surface. There is no evidence of malignancy. The anatomical pathology result suggested a splenic pseudocyst. According to the medical record, the patient visited the clinic four times for post-operative control. Despite receiving the vaccines (Haemophilus influenza and Streptococcus pneumonia) prior to the splenectomy procedure, the patient frequently complained of fever.

## 3 Discussion

A rare disease with an incidence rate of 0.07–3 % is splenic cyst, which is more common in women. Typically, splenic cysts are categorized as parasitic or non-parasitic based on their etiology. Echinoccocal parasitic cysts account for 50–80 % of splenic parasitic cysts. In rare cases, 0.5–4 % of cases of hydatid disease can result in a parasitic cyst on the spleen [12]. Non-parasitic cysts are categorized as primary (true) or secondary (false) based on whether or not they have a cellular epithelial lining. Primary (true) splenic cysts, according to Fowler and Martin, have an epithelial lining. Secondary (false) cysts do not have an epithelial lining. The most prevalent non-parasitic cyst type is an epithelial cyst, which accounts for about 10 % of all splenic cysts [13], [14], [15], [16].

Of all splenic cysts, false ones (secondary) make up about 80 % of the total. The most frequent causes of secondary splenic cysts are trauma, splenic infarction, disorganized hematoma, splenic abscess, and intrasplenic pancreatic pseudocyst. Secondary splenic cysts that are caused by infection, most often mononucleosis or tuberculosis [17]. True splenic cysts, on the other hand, make up just 20 % of all splenic cysts and can be brought on by a tumor (hemangioma and lymphangioma), an infection (most commonly hydatid disease), or a congenital condition (primary epithelial cysts) [1]. A single unilocular lesion with thin, smooth walls and no peripheral calcification characterizes a true cyst. Most true splenic cysts come from the epithelium and contain embryonic epithelial cells from nearby structures. The high production of tumor antigens such as carcinoembryonic antigen (CEA), CA19-9, CA125, and CA50 is associated with true splenic cysts, which can be found in both the serum and the cyst contents [18].

Spleen cysts appear to be a coincidental finding and are usually asymptomatic unless they grow to a significant size and cause symptoms such as abdominal discomfort, splenomegaly, or fever. When a cyst grows larger than 5 cm, it becomes symptomatic, and symptoms worsen when it grows larger than 8 cm [17], [18], [19]. The signs and symptoms of a splenic cyst can include an abdominal mass, less frequently in the epigastrium, and most frequently in the left hypochondrium. Pain may be the initial clinical symptom in some cases. Dyspepsia and constipation are caused by colon pressure, and dyspnea can occur as the left diaphragm is pushed. According to some sources, a spleen cyst can cause hematemesis. When there is a rupture, the clinical findings may include massive hemoperitoneum and anaphylactic reactions. Patients may present late after the cyst has ruptured because most cases are asymptomatic. Symptomatic splenic cysts require definitive treatment [20], [21], [22].

The majority of splenic cysts are symptomatic, making diagnosis challenging. Imaging in conjunction with immunological tests may be able to solve diagnostic issues. Radiological findings ranging from cystic to solid, unilocular to multivesicular cyst. Calcification of the cyst wall is possible. Immunological tests like hemagglutination inhibition (HAI), enzyme-linked immunosorbent assay (ELISA), and Western blot can help confirm a diagnosis. If the cyst is intact, calcified, or sterile, an immunological test may be negative or insufficient for a definitive diagnosis [20], [21], [22].

A splenic cyst can cause a life-threatening situation due to a spontaneous or traumatic rupture, infection, and bleeding of the spleen, which may lead to anaphylaxis [13]. Alcohol sclerosing injection, marsupialization, partial or complete splenectomy, and percutaneous aspiration can all be used to treat a splenic cyst. Percutaneous aspiration and marsupialization carry the risk of cyst recurrence and, in the worst-case scenario, splenectomy [19]. Sclerosing agents, such as alcohol and tetracycline, reduce the risk of recurrence. The standard treatment is a total or partial splenectomy, both of which have advantages and disadvantages. To reduce intracystic pressure, aspiration and puncture of the cyst can be used to drain the fluid, although splenectomy is preferred. Although partial splenectomy can result in recurrence and post-operative hemorrhage, total splenectomy should be avoided due to the risk of sepsis, especially in children [12]. To prevent post-splenectomy infection, which might result in death in the healing period, spleen preservation should always be tried. Spleen preservation procedures include partial splenectomy, cyst enucleation, cyst deroofing with omentoplasty, and external drainage. Laparoscopic splenectomy is another option available at centers for advanced laparoscopy. However, some authors think that laparoscopic splenectomy carries a greater risk of anaphylactic shock and intra-peritoneal dissemination [23], [24], [25], [26].

Only two patients have been recorded in the medical record and treated in our tertiary general hospital's digestive surgery division in the last 22 years, which is the referral center in West Java, a province in Indonesia with a population of 49.94 million. This condition shows that a splenic cyst is a very rare occurrence. The difference between those two patients in this case is the surgical procedure. A partial splenectomy, or peri-cystic splenectomy, was performed on the first patient because the cyst was single and located in the superficial surface of the spleen. Whereas a total splenectomy was performed on the second patient because the cysts were multiple, located deep in the spleen, and covered with dense parenchyma of the spleen. The distinction between these two procedures also has an impact on the length of stay in the hospital. The first case has a longer length of stay than the second. This condition is caused by the production of drainage fluid, which contains more product than the second for the first few days following surgery. Another distinction is the risk of infection. When compared to the first patient, the second patient has a higher risk of infection. In this case, the second patient had already been vaccinated to avoid the occurrence of OPSI (overwhelming post-splenectomy infection), but the second patient complained that the patient had more fever and flu than the first patient, who did not receive the vaccine after the surgery. This case demonstrated that conservative treatment is more favorable for splenic cysts if possible.

## 4 Conclusion

Splenic cysts are uncommon. As a rare disease, diagnosing a splenic cyst is still challenging because only a few cases have been reported. Nonetheless, proper management is still required because there is a risk of rupture, which can lead to complications such as peritonitis and anaphylactic reactions. Splenectomy was once considered the gold standard for splenic cysts, but it is now being called into question, given the contemporary preference for conservative surgery, especially for a benign pathology, and awareness of postoperative risks.

## Consent

Before this case report and the accompanying images could be published, the patient's written informed consent had to be obtained. Upon request, a copy of the written consent may be reviewed by the journal's editor-in-chief.

## Provenance and peer review

Not commissioned, externally peer-reviewed.

## Ethical approval

No ethical clearance was required.

## Funding

The author(s) received no funding for this case report.

## Guarantor

The guarantor of this report is Kiki Lukman.

## Research registration number

Not applicable.

## CRediT authorship contribution statement

**Kiki Lukman:** Conceptualization, Visualization, Supervision, Funding acquisition. **Bambang Am Am Setya Sulthana:** Investigation, Data curation, Resources. **Deny Budiman:** Investigation, Data curation, Resources. **Prapanca Nugraha:** Writing – original draft, Writing – review & editing, Project administration.

## Declaration of competing interest

The authors affirm that they have no known financial or interpersonal conflicts that would have appeared to have an impact on the case report presented.
